# Prospective, non-randomised, open-label pilot trial assessing feasibility, safety and treatment success of acupuncture in children with functional constipation: ACU-PILOT study protocol

**DOI:** 10.1136/bmjopen-2025-109425

**Published:** 2025-11-11

**Authors:** Michelle N Bloem, Desiree F Baaleman, Ilan J N Koppen, Arine M Vlieger, Fleur de Lorijn, Stephen Birch, Max Nieuwdorp, Marc A Benninga

**Affiliations:** 1 Department of Pediatric Gastroenterology, Emma’s Children Hospital, Amsterdam UMC, Locatie AMC, Amsterdam, the Netherlands; 2 Amsterdam Gastroenterology Endocrinology Metabolism Research Institute, Amsterdam UMC, Amsterdam, the Netherlands; 3 Amsterdam Reproduction and Development Research Institute, Amsterdam UMC, Amsterdam, the Netherlands; 4 Department of Pediatrics, St Antonius Hospital, Nieuwegein, the Netherlands; 5 School of Health Sciences, Kristiania University College, Oslo, Norway; 6 Department of Internal Medicine, Amsterdam UMC, Locatie AMC, Amsterdam, the Netherlands

**Keywords:** Motility disorders, Acupuncture, Functional bowel disorders, Paediatric gastroenterology, Child

## Abstract

**Introduction:**

Functional constipation (FC) is prevalent among children and often persists despite standard pharmacological treatment with oral laxatives. Many parents turn to complementary therapies, including acupuncture, which has been shown to relieve symptoms in adults with FC. However, studies in children with FC are scarce and have important limitations. This study will evaluate the feasibility, safety and potential efficacy of acupuncture for children with FC.

**Methods and analysis:**

Prospective, non-randomised, open-label pilot study in children with FC (6–18 years). Participants will undergo eight acupuncture sessions over 10 weeks. Concurrent pharmacological treatment with polyethylene glycol (≥0.2 g/kg/day) will continue as initiated prior to enrolment. The primary endpoint is feasibility, defined by an attrition rate of ≥70%. Secondary feasibility endpoints include consent rate, patient/parent satisfaction and personnel requirements. Safety will be assessed by systematic monitoring of adverse events. Efficacy endpoints include treatment success, defined as no longer meeting the Rome IV criteria for FC at the end of the intervention period, as well as defecation frequency, stool consistency, painful defecation, faecal incontinence frequency, abdominal pain, medication use and quality of life, based on a previously published core outcome set.

**Ethics and dissemination:**

Ethical approval was provided by the Medical Ethics Committee of Amsterdam UMC (Netherlands; NL87083.018.24). Results will be published in peer-reviewed journals and presented to scientific and consumer audiences.

**Trial registration numbers:**

NCT06836362 and NL-OMON57236.

**STRENGTHS AND LIMITATIONS OF THIS STUDY**

- The open-label design and continuation of standard pharmacological treatment enhance the applicability of findings to current clinical practices worldwide.
- Strict inclusion criteria requiring polyethylene glycol as primary pharmacotherapy and allowing limited stable co-medication ensure greater uniformity within the study group but may limit the generalisability of results to children receiving other treatments for functional constipation, such as other medications or trans-anal irrigation.
- Acupuncture is administered according to a semi-standardised protocol, combining a core set of acupoints with individualised point selection based on clinical assessment.
- Feasibility endpoints, including attrition rates and satisfaction, will inform the potential design of a future randomised controlled trial.
- While efficacy will be measured, the absence of a control group limits the assessment of acupuncture’s clinical efficacy.

## Introduction

 Functional constipation (FC) is a common disorder in children and adolescents with a worldwide pooled prevalence of 9.5%.^1^ It is characterised by infrequent, painful, hard stools and is often accompanied by faecal incontinence and abdominal pain.^2^ FC is a clinical diagnosis based on history and physical examination and is defined according to the Rome IV criteria (table 1).^3^ It has a significant psychological impact on the quality of life of children and their parents.^4 5^ In addition, FC, often accompanied by symptoms of faecal incontinence, can negatively affect the healthy psychosocial development of children and lead to feelings of frustration among parents.^6^

**Table 1:** Eligibility criteria for participants

Inclusion criteria	Exclusion criteria
6–18 years of age	Irritable bowel syndrome (IBS)*
Insufficient symptom management† despite ≥3 months of medical management (including education, non-pharmacological advice and laxatives) by a physician	Organic causes of constipation (eg, spina bifida or Hirschsprung disease)
Are treated with PEG (minimum dose of 0.2 g/kg/day) for ≥1 month prior to inclusion in the study	Concomitant use of drugs not intended for FC treatment that are known to affect gastrointestinal motility
Meet the modified Rome IV criteria for children and adolescents with FC^3^‡:	Previously received acupuncture for constipation or currently participating in another clinical trial
1) ≤2 spontaneous bowel movements (SBMs) per week (SBM *defined as* a bowel movement that occurs in the absence of laxative, enema or suppository use in the preceding 24 hours)	Significant chronic health conditions requiring specialty care (eg, cardiac, renal or metabolic diseases) that could impact the child’s ability to participate or confound the results of the study
2) History of excessive stool retention	Recurrent or unexplained fevers
3) History of painful or hard bowel movements	Pregnancy
4) History of large-diameter stools	Smoking
5) Presence of a large faecal mass in the rectum	Severe needle-related anxiety
6) ≥1 episode/week of incontinence after the acquisition of toileting skills	History of abdominal surgery except appendectomy or hernia repairs
7) History of large-diameter stools that may obstruct the toilet in toilet-trained children	Unintentional weight loss ≥5% of body weight within the last 3 months
Written informed consent (parents/guardians and children ≥12 years)	Clotting disorders or recent history of thrombocytopenia
	Immunocompromised children
	Established diagnoses of autism spectrum disorders
	Established major psychiatric disorders or a history of abuse
	Rash or active local infection over an acupuncture point

* Rome IV criteria (fulfilled for ≥2 months before diagnosis) for IBS in children and adolescents include all of the following: (1) Abdominal pain at least 4 days per month associated with one or more of the following: (a) Related to defecation. (b) A change in frequency of stool. (c) A change in the form (appearance) of stool. (2) In children with constipation, the pain does not resolve with resolution of the constipation (children in whom the pain resolves have functional constipation, not IBS). (3) After appropriate evaluation, the symptoms cannot be fully explained by another medical condition.

† Insufficient symptom management is defined as the presence of ≥1 of the Rome IV criteria for FC *despite* medical management by a physician.

‡ Defined as meeting ≥2 of the following criteria during the 2-week run-in period despite receiving treatment with PEG (min. 0.2 g/kg/day).

FC, functional constipation; PEG, polyethylene glycol.

From a global health perspective, the burden of FC on healthcare and healthcare costs is significant. Due to the protracted nature of childhood FC, it accounts for up to 25% of visits to paediatric gastroenterologists in the USA.^7^ Annual healthcare costs related to FC vary between studies but are estimated to be three times higher in children with FC than for children without constipation, accounting for an additional cost of up to $3.9 billion/year in the USA alone.^8 9^

Initial treatment consists of non-pharmacological and pharmacological interventions.^10^ Non-pharmacological interventions involve education and demystification, toilet training (with a reward system), and keeping a defecation diary. First-line pharmacological treatment involves oral osmotic laxatives, preferably polyethylene glycol (PEG).^10^ Osmotic laxatives are considered safe, but adherence to PEG is generally low.^11^ In rare cases, surgical interventions such as antegrade continence enemas or colonic resections with or without diverting ostomy are performed as a last resort.^12^

Despite these different interventions, approximately 40% of children with FC referred to a paediatric gastroenterologist remain symptomatic after 5 years, and even after 10 years of treatment, 20% of children still suffer from FC symptoms.^10^ Unresolved, chronic symptoms may lead to a search for alternative treatment strategies. A Dutch study in 2008 reported that almost 40% of parents of paediatric patients with functional and organic gastrointestinal diseases use complementary and integrative medicine for their child.^13^ However, evidence to support such practices in children is often lacking or of low quality. Therefore, there is an urgent need for high-quality research in this field to uncover both potential benefits and possible risks related to practices that have not yet been studied adequately but are currently being performed on children worldwide.

Acupuncture is an ancient therapeutic technique that involves stimulating specific acupuncture points on the body, known as acupoints, aiming to modulate physiological responses. Stimulation of acupoints can occur through several mechanisms, including non-invasive paediatric techniques such as Shonishin, which involves gentle tapping, rubbing or pressing of the skin to stimulate acupoints, laser techniques (laser acupuncture) or the insertion of fine needles (manual acupuncture) potentially accompanied by electrical stimulation (electro-acupuncture).^14 15^ In China, acupuncture has been used for thousands of years and in recent times a considerable number of studies have been performed on acupuncture for the treatment of disorders of gut-brain interaction in adults, including FC.^16 17^ Results of several randomised controlled trials (RCTs) in adults indicate that acupuncture may be effective in treating FC by increasing stool frequency, improving stool consistency, alleviating constipation symptoms and improving quality of life.^16^ However, these studies have used a variety of study designs, are often of poor quality and have mostly been performed in China.^16^

Although the exact mechanisms of acupuncture are not fully elucidated, acupuncture is thought to potentially improve FC symptoms via multiple mechanisms, including regulation of both the enteric nervous system and the central nervous system, thereby improving gut motility.^16^,^18^ The regulatory system linking the central and enteric nervous system to the peripheral intestinal functions is referred to as the brain-gut axis. This complex bidirectional system includes the parasympathetic and sympathetic nervous system, endocrine and immune factors, and the gut microbiota, all regulating gastrointestinal homeostasis.^19^,^21^ The vagal nervous system, as main component of the parasympathetic nervous system, is important in the regulation of the gastrointestinal system,^21^ exerting both excitatory and inhibitory control over the gastrointestinal tract.^18^ The role of the sympathetic nervous system is mainly inhibitory^18^ and it is likely that its activation via release of stress hormones such as epinephrine, norepinephrine and cortisol contributes to a dysregulation of gut motility.^22^ Some studies in animals and humans have suggested a role for acupuncture via regulation of the enteric nervous system, although mostly in the context of visceral pain management.^23^,^29^

Although several researchers have attempted to elucidate possible mechanisms of action of acupuncture, it is challenging to pinpoint a single mechanism. This may partially be due to the diversity of acupuncture techniques, variations in local receptor types and densities at different points (including neuroreceptors, mechanoreceptors or receptors on immune and interstitial cells) and differences in which receptors are activated depending on the depth and method of needling.^28^,^31^ Any potential effects of acupuncture are most likely explained by a combination of various factors.

To date, although promising, published data on the effects of acupuncture on FC symptoms in children are limited^32^,^34^ and published reports in adults have important limitations to take into consideration when interpreting these results.^16^ Anders *et al* applied a single acupuncture treatment with fixed indwelling acupuncture needles in 10 children (0.5–15 years) with hospital-induced constipation (defined as no stool within 72 hours after hospital admission) before conventional therapy with laxative suppositories was initiated.^33^ Patients, parents and nurses were encouraged to perform needle stimulation at acupuncture point LI-11 by means of massage for 2–3 min two times an hour. After stimulation of acupuncture point LI-11, defecation occurred within 2 hours in all patients without the need for administration of conventional laxative treatment. Broide *et al* included 27 constipated children (aged 3–13 years old) administering five weekly sham acupuncture sessions, followed by ten weekly true acupuncture sessions on LI-2, LI-4 and/or ST-36.^32^ A significant effect was reported of true acupuncture sessions on defecation frequency compared with baseline. The authors hypothesised that the effect of acupuncture may have been related to the release of opioid peptides. In their study population, they found a low basal plasma panopioid activity in constipated children compared with a healthy control group, which slowly increased during the study period. Abd El Azeem *et al* included 40 constipated children (aged 5–15 years old) in an RCT, comparing standard non-pharmacological interventions in combination with either laser acupuncture or lactulose.^34^ Laser acupuncture was performed at ST-25, ST-36, ST-37, BL-25 and LI-11 three times weekly for 4 weeks. A significant increase in bowel movement frequency in both groups (four times and three times a week in the acupuncture vs the lactulose group, respectively) was reported, in favour of the laser acupuncture group, as compared with pretreatment frequency (two times a week in both groups). Notably, these effects persisted 3 months after discontinuation of treatment, suggesting acupuncture’s potential impact on FC.

In summary, the results of three studies performed in children with constipation have shown promising results.^32^,^34^ Clinical findings of randomised sham-controlled trials in adults also suggest a positive effect of acupuncture on FC symptoms.^16^ However, currently, there is not one uniform, well-established acupuncture treatment protocol for patients with FC, since trial designs in adults with FC have used varying protocols with different outcome measures.^16^ Moreover, although published data on the effectiveness of acupuncture for patients with FC are promising, there are important limitations to take into consideration when interpreting these results. First and foremost, the quality of currently published research is often poor.^16^ In recent guidelines on FC in adults, the European Society of Neurogastroenterology and Motility has recommended that better-designed trials are necessary before the utility of acupuncture in constipation can be evaluated.^35^ Another major limitation of the currently available evidence is that most of these data come from Chinese studies and publication bias may affect the validity and reliability of these data. Therefore, there is an urgent need to assess the applicability of acupuncture in Western care settings, with methods adhering to internationally accepted research standards. In addition, there is a general lack of studies with acupuncture in children with FC and it is uncertain if children respond similarly to adults with FC, since the pathophysiological mechanisms underlying FC may vary between children and adults.^12^

Thus, the primary objective of this pilot study is to assess the feasibility of acupuncture in children with FC using an intervention protocol which was based on the available literature and expert opinion.^1632^,^34 36^ We will test the integrity and feasibility of this intervention protocol; if deemed feasible, we will conduct an RCT using this intervention protocol. Secondary objectives are to assess safety, tolerability and potential clinical efficacy of acupuncture in children with FC. We intend to collect sufficient information on endpoints that are important to enable the design of a future RCT.

## Methods

### Study design

**Figure 1:**
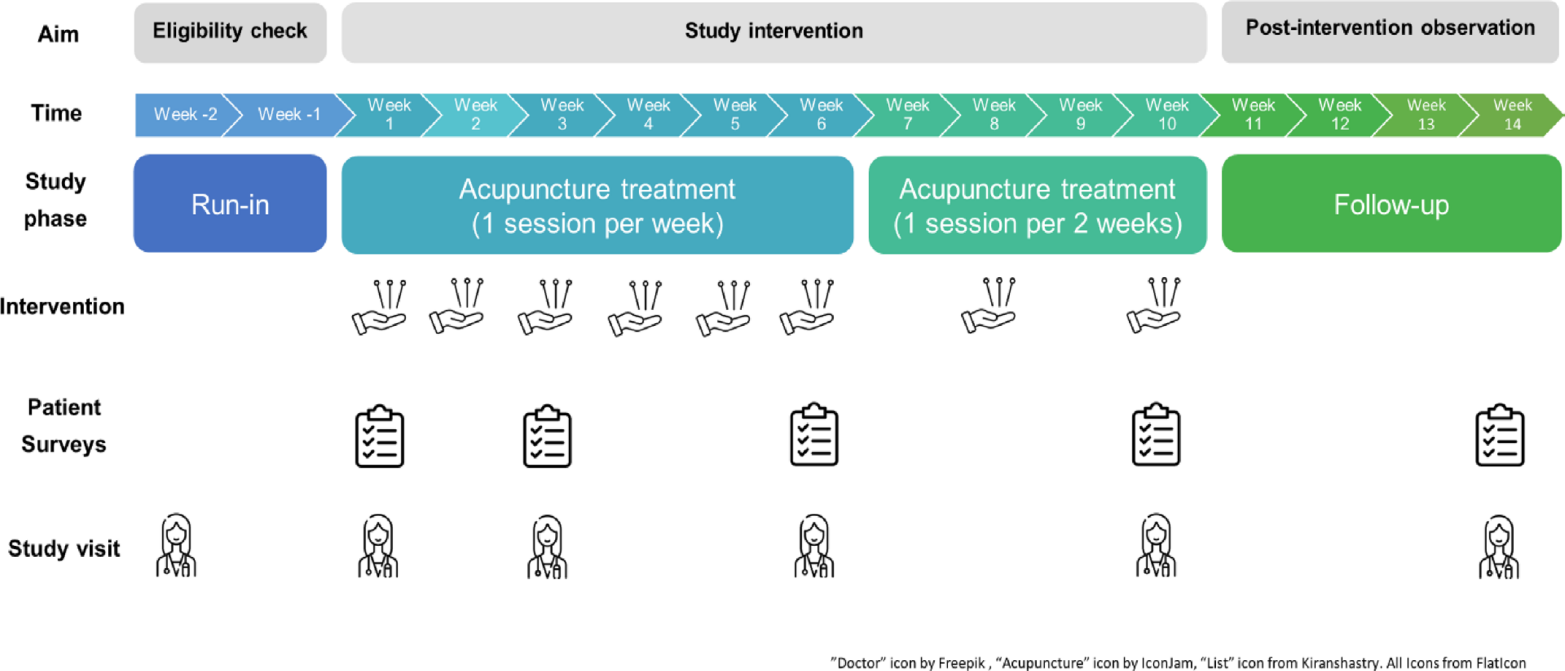
Schematic depiction of study design. Participants will receive eight acupuncture sessions over 10 weeks. At each session, symptoms and adverse events over the previous week will be recorded. Participants will complete surveys at five time points, including the validated PedsQL 4.0 Generic Core Scale and 3.0 Gastrointestinal Symptoms Scale and module, and questions monitoring adverse events, treatment satisfaction and school absence. After the baseline eligibility check, participants will visit the research nurse for five study visits, allowing for assessment of primary and secondary endpoints. The study visits at t=0, t=3, t=6 and t=10 will take place after the acupuncture sessions.

This is a non-randomised, uncontrolled, open-label longitudinal interventional pilot study, designed in line with recommendations from the Rome foundation paediatric subcommittee on pharmacological clinical trials in children with FC.^37^ The protocol is designed in alignment with the Standards for Reporting Interventions in Clinical Trials of Acupuncture.^38^ Expected recruitment phase is June 2025 to January 2026. A schematic depiction of the study design is shown in figure 1.

### Participants

Research subjects will be selected based on a diagnosis of FC according to the Rome IV criteria (table 1).

### Sample size calculation

This feasibility pilot study evaluates an intervention protocol not previously studied. A sample size of 18 patients was deemed sufficient to assess the primary objective, based on consultation with a statistician.^39 40^ The sample size calculation considers a significance level of alpha=0.05, power of 0.8, estimated feasibility of 0.9, and a lower bound of 0.7 for study exclusion. This sample size minimises patient exposure while providing enough data to inform the future RCT.

### Recruitment procedure

Eligible participants will be informed about the study by their treating physician, who will seek consent to contact them for further details. The researcher will then provide the Patient Information Sheet and Consent Form. Participants will have 2 weeks to consider participation, with follow-up calls to answer any questions. If consent is given, the study team will finalise the consent process at the next clinic visit. The age and gender of eligible participants who have been informed about the study but choose not to participate will be registered, along with the reason for not participating, if provided. Recruitment will occur mainly in the outpatient clinic, with additional flyers distributed online and in general practitioners’ offices. Interested individuals can contact the researcher directly. Patients who previously expressed interest in an intervention study during an ongoing survey at Amsterdam UMC will also be contacted.

### Informed consent

In children <12 years of age, informed consent is obtained from parents and verbal assent is obtained from the child. In children ≥12 years, written informed consent is obtained from the parents/guardians and the child.

### Patient and public involvement

The semi-standardised acupuncture treatment protocol in this study has been co-designed in collaboration with acupuncturists in a global network. Prior to the study, a survey was conducted to assess the willingness of children aged 8 years and older with functional gastrointestinal disorders (FGID), as well as parents of children of all ages with FGID, to participate in acupuncture-related research. The survey was anonymous and focused on understanding participants’ readiness to engage in research. While the views of patients and the public have informed some aspects of the study design, no direct patient or public involvement was actively incorporated into the development of this protocol.

### Study schedule

The study lasts 16 weeks, with 10 visits and eight acupuncture treatments.

#### Visit 1: eligibility check (t=−2)

Patients will be assessed for eligibility based on inclusion and exclusion criteria. Baseline data, including anthropometrics, will be recorded. Patients will receive instructions on defecation diaries and escape medication usage. A 2-week baseline run-in period follows, where participants continue PEG treatment (min. 0.2 g/kg/day). Children who meet the modified Rome IV criteria for FC (adjusted for the duration of the run-in period) will be enrolled to receive acupuncture (table 1).^3^

#### Visit 2: screening and first acupuncture treatment (t=0)

After the run-in period, eligibility is confirmed based on the diary information, according to the modified Rome IV criteria. A bowel clean-out regimen (bisacodyl or enemas) is initiated for 2–3 days (box 1). On successful bowel clean-out, the first acupuncture session begins.

Box 1 **Escape medication**

**Bisacodyl**

<11 years of age: 5 mg per dose, once daily (oral/rectal).^*^

≥11 years of age: 10 mg per dose, once daily (oral/rectal).^*^

**Sodium docusate (enema)**

6–12 years: 50–120 mL per dose, once daily.

≥12 years: 120 mL per dose, once daily.

**Sodium phosphate (enema)**

6–18 years of age: 2.5 mL/kg per dose, once daily (maximum 133 mL/dose).

^*^Patients are initially allowed an administration route of choice. However, if 24 hours after oral administration of bisacodyl no defecation has occurred, on the second day, the dose needs to be administered rectally. The third day, an enema is required. Enemas are administered at body temperature.

#### Visit 3–9: acupuncture treatment

Patients receive eight acupuncture sessions over 10 weeks (one per week for 6 weeks, then one every other week for 4 weeks). Acupuncture sessions are conducted by one of two acupuncturists, with a physician or research nurse present. At each acupuncture session, the acupuncturist records the patient’s bowel habits, gastrointestinal symptoms and the use of escape medication during the period between sessions, based on the patient’s symptom diary. At weeks 3, 6 and 10, adverse events (AEs) are evaluated.

#### Follow-up

During the 4-week acupuncture-free follow-up, escape medication can be used under the same conditions as during the run-in period. If required for the eighth time, conventional treatment adjustments are proposed and documented.

#### Visit 10: end of study visit (t=14)

Four weeks after the final acupuncture session, an end-of-study visit is conducted. AEs are assessed, and weight/height is recorded.

#### Questionnaires and diaries

Patients complete questionnaires on treatment satisfaction, AE, PedsQL and school absence at specified visits. Symptom diaries are maintained daily from the run-in up to the follow-up visit (table 2).

**Table 2:** Study procedures

	T=−2 Eligibility screening	T=0 Screening patient	T=2	T=4	T=6	T=8	T=10	T=14
Research visit (duration in minutes)	X (60)	X (30)		X (30)		X (30)		X (30)
Check for exclusion criteria	X	X						
Informed consent	X							
Rome IV criteria FC assessment	X	X		X		X		X
Acupuncture								
−1 sessions/week			X*	X*	X*			
−1 session/every other week						X*	X*	
Symptom diary	X†	X†	X‡	X‡	X‡	X§	X§	X¶
Adverse events								
Research nurse			X		X		X	X
Acupuncturist		X	X	X‡	X	X‡		
Questionnaires								
Quality of life		X			X		X	X
School absenteeism		X						X
Satisfaction with treatment					X		X	X

* Patients will receive eight acupuncture treatments during 10 weeks (one session per week during 6 weeks, followed by one session every other week during 4 weeks) between t=0 and t=10.

† Patients will fill out the symptom diary daily for the first 2 weeks.

‡ Symptoms over the prior week will be recorded weekly at each acupuncture session.

§ Symptoms over the prior 2 weeks will be recorded fortnightly at each acupuncture session.

¶ Symptoms over the prior 4 weeks will be recorded at the closing session.

FC, functional constipation.

### Medication and co-interventions

Participants will continue their prestudy PEG dosage (≥0.2 g/kg/day) and non-pharmacological constipation treatments as previously prescribed by the medical team in charge. Concomitant use of laxatives other than PEG and other than escape medication (such as other osmotic or stimulating laxatives, eg, magnesium hydroxide, sodium picosulfate) is permitted if participants have been treated for at least 3 months prior to study initiation, as it is considered unethical to require discontinuation of such established therapy. The use and dosage of these co-medications will be documented. Current medication unrelated to defecation (eg, asthma medication) should be maintained. Initiation of new FC treatments or PEG dose increases during the intervention is prohibited. PEG dose reductions, if prescribed, will be documented. New medical treatments during the study will be assessed for impact, with potential withdrawal if necessary. Any PEG dose increase after the final acupuncture session will be recorded during follow-up.

### Escape medication

As is common in paediatric research settings, if a child does not defecate for 72 hours or experiences faecal impaction symptoms, escape medication (bisacodyl or enemas) will be given daily for up to a maximum of 3 days.^37 41 42^ If ineffective, the participant will be examined and withdrawn if faecal impaction is present. Dosage regimens adhere to the European Society for Pediatric Gastroenterology Hepatology and Nutrition^10^ and the Dutch Pediatric Formulary (https://www.kinderformularium.nl/) recommendations (box 1). Patients requiring disimpaction eight or more times during the intervention are considered treatment failures and will be withdrawn.

### Treatment

**Figure 2:**
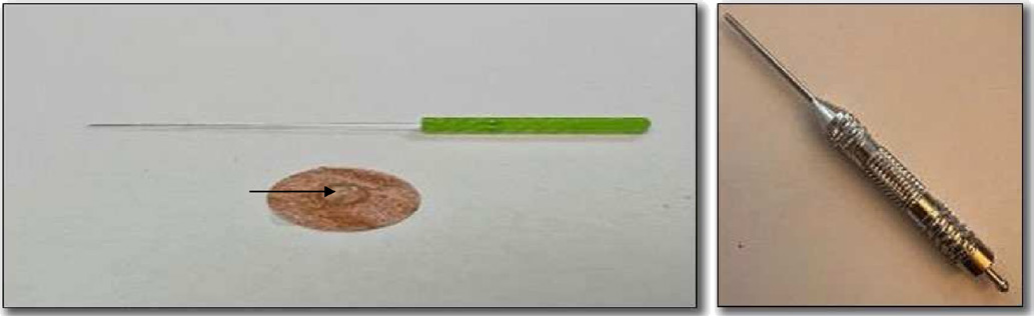
Acupuncture tools. Left, 0.14×30 mm Seirin acupuncture needle (above) and Seirin Pyonex press needle (below). Arrow points to 0.2×0.3 mm needle. Right, the Tsumoshin tool, a blunt-tipped non-penetrating needle with a rounded millet-seed-like point used for pressing the body surface.^15^

This study evaluates acupuncture administered by a certified acupuncturist using Seirin Pyonex press needles (0.2×0.3 mm for children <9 years, 0.2×0.6 mm for those ≥9 years) and 0.12–0.16×30 mm Seirin acupuncture needles (3B Scientific GmbH, Hamburg, Germany), all CE-certified (CE0123) medical devices used as intended. Acupuncture treatment combines four treatment techniques adapted to age: non-penetrating Shonishin techniques (stroking, pressing and tapping; online supplemental appendix 1), non-penetrating Tsumoshin technique (acupoint pressing; figure 2, online supplemental appendix 2), inserted needles with an applicator guide tube (minimising sensory stimulation) and Pyonex press needles (figure 2).

**Figure 3:**
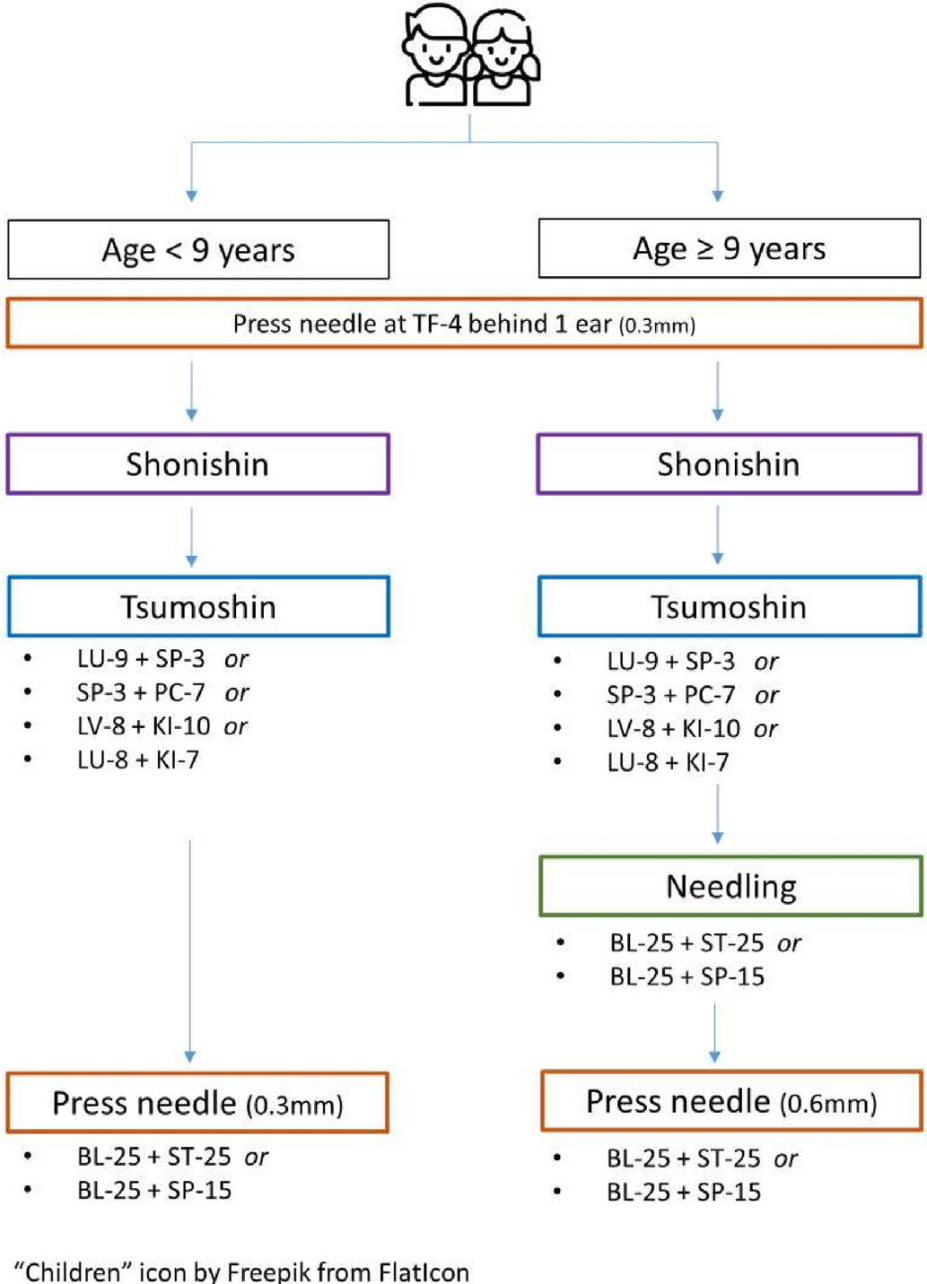
Acupuncture treatment decision tree according to age groups—session 1. All needles and press needles are placed bilaterally, unless stated otherwise. Shonishin: a Japanese non-penetrating treatment method using techniques of stroking, pressing and tapping, applied down the back and back of the legs, down the abdomen and anterio-lateral edges of the legs and down the arms.^15^ Tsumoshin: non-penetrating technique of acupoint pressing using the Tsumoshin tool, a blunt-tipped needle with a rounded millet-seed-like point used for pressing the body surface.^15^

**Figure 4:**
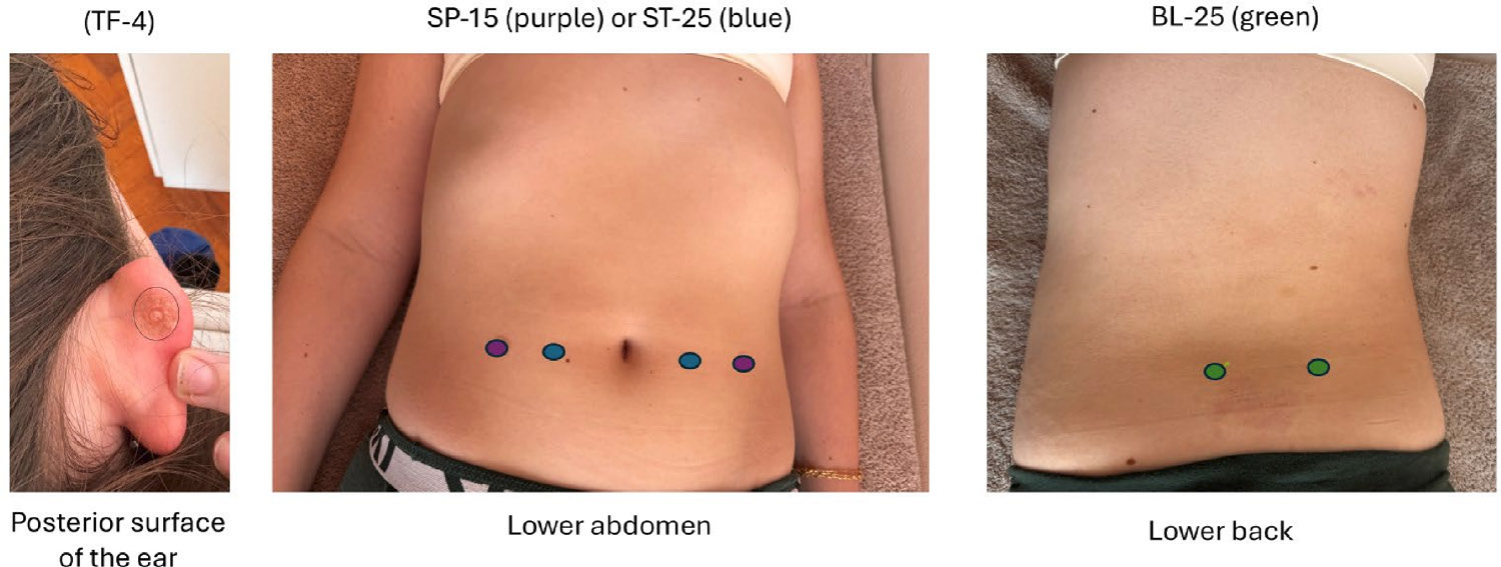
Localisation of main acupoints targeted with (press-tack) needling at every acupuncture session. Pictures taken with patient consent for publication in a medical journal. Coloured dots (middle and right images) were added for schematic illustration. The press-tack needle placed at the Ear Shen Men (TF-4) on the posterior surface of the right auricle is highlighted with a black circle to enhance visibility.

Patients are treated in both supine and prone positions, with acupoint selection based on age and acupuncturist evaluation. Treatment consists of standard and optional components (figure 3) and is discontinued if needle positioning poses a safety risk or if the child experiences excessive fear or stress. All patients are first treated with non-penetrating Shonishin and Tsumoshin techniques per traditional Japanese acupuncture principles (online supplemental appendices 1 and 2).^15^ Press needles are placed at BL-25 (Dachangshu) and TF-4 (behind the Ear ShenMen) in all sessions, with either ST-25 (Tianshu) or SP-15 (Daheng) selected based on acupuncturist’s evaluation (figure 4). Acupoints were selected based on literature and expert consensus.^16 34 36^

Treatment progresses through an initial session (figure 3), followed by three sessions incorporating additional points (online supplemental figure 1) and four further expanded sessions (online supplemental figure 2), further detailed in the following section. Step-ups may be delayed or advanced based on clinical evaluation, with reasons documented. The acupuncturist may select optional acupoints relevant to defecatory issues, limited to a predefined set (figure 3, online supplemental file 1/2). These were determined through literature review and a survey of 39 experienced acupuncturists across Europe, Asia, Oceania and North America specialising in Shonishin/Japanese Meridian Therapy, with 29 providing input on techniques and acupoint selection. Standard and optional acupoint locations are detailed in figure 4, online supplemental appendix 3.

### Acupuncture

#### Session 1

All children receive non-penetrating Shonishin techniques (stroking, pressing, tapping) and a unilateral press-tack needle behind the ear at TF-4 (ShenMen) (figure 3). Light pressure is applied using Tsumoshin for one of four root pattern treatments, selected based on Traditional Japanese Medicine principles after history and examination (figure 3, online supplemental appendix 1). In children <9 years, treatment concludes with bilateral 0.3 mm Pyonex press-tack needles at BL-25 and either ST-25 or SP-15, left indwelling and replaced at the next session. In children ≥9 years, needling at BL-25 and ST-25 or SP-15 bilaterally is followed by 0.6 mm Pyonex press-tack needles at the same points, left indwelling and replaced at the next session. Press-tack needles dislocated >48 hours after placement or purposefully removed at any time are not replaced but documented.

#### Sessions 2–4

In children <9 years; before placing press-tack needles, BL-25 and ST-25 or SP-15 are needled bilaterally for 5 min using Seirin needles, followed by bilateral Pyonex press needles (0.3–0.6 mm) based on pain response and prior treatment (online supplemental figure 1). In children ≥9 years, two additional points (selected from LI-11, ST-27, SP-14, SP-6) are needled per session, totalling six needles (online supplemental figure 1).

#### Sessions 5–8

In children <9 years, additional needling (1–3 points) is introduced based on persistent Rome IV criteria for FC, selecting from LI-11, ST-27, SP-14 and SP-6 (online supplemental figure 2). Final sessions involve 5–7 needles. Five press-tack needles (0.3–0.6 mm) remain standard at BL-25, ST-25 or SP-15 bilaterally, and behind the ear at TF-4 unilaterally (figure 3C). In children ≥9 years, up to four additional points (TB-6, CV-12, KI-6, ST-36) are selected based on persistent Rome IV criteria for FC, increasing to a maximum of 10 needles in final sessions. Acupuncture points and rationale are documented per session. Five press-tack needles (0.6 mm) remain standard at BL-25, ST-25 or SP-15 bilaterally, and TF-4 unilaterally (online supplemental figure 2).

### Safety monitoring

Acupuncture is generally well-tolerated, with infrequent and mild AEs reported in paediatric studies.^16 43 44^

#### Risks and benefits

All participants will receive standard care in addition to acupuncture. Acupuncture is considered safe, with a ‘negligible risk’ classification according to the NFU (Dutch Federation of University Medical Centers) guidelines. No additional participant insurance is required as no additional risks are expected. Participants will receive free acupuncture treatment by certified acupuncturists, with reimbursement for travel and parking costs.

#### Adverse events

AEs are defined as any undesirable experience occurring to a subject during the study and considered related to acupuncture treatment. Predefined AEs—including needle-related pain, local irritation, haematomas, headache, syncope, sedation and neuropathy—will be assessed weekly via questionnaires. All AEs observed by the investigator or reported by participants will be documented, including severity (mild, moderate or severe) and relationship to treatment (unrelated, possibly, probably or definitely related). Given the nature of acupuncture, serious AEs are not expected.

AEs occurring from the first study-related procedure until 3 months postintervention will be recorded in the case report form and monitored until resolution or stabilisation. Follow-up may require additional tests, medical procedures or referral to a physician or specialist.

#### Premature termination or withdrawal of subjects

If premature study termination occurs, all available data will be published. In accordance with section 10, subsection 4, of the Medical Research Involving Human Subjects Act, the sponsor will suspend the study if continuation is deemed to jeopardise participant health or safety. The accredited Medical Ethics Committee (METC) will be notified without undue delay, and the study will remain suspended until a positive decision is obtained. Participants will be informed accordingly. Participants may withdraw at any time without consequences. The investigator may also withdraw participants for urgent medical reasons. To maintain the target sample size, an additional participant will be recruited if withdrawal occurs during the run-in period. If withdrawal is unrelated to the study (eg, relocation), a new participant will be recruited. Withdrawn participants will be followed up via online questionnaires, with assessments conducted via telephone if needed.

#### Data Safety Monitoring Board

Given the low-risk nature of the study, with only eight acupuncture sessions over 10 weeks and no expected serious adverse effects, a Data Safety Monitoring Board is not required.

### Outcomes

#### Primary outcome

##### Feasibility

Attrition rate, defined as the percentage of participants completing the study protocol. The intervention will be considered feasible if ≥70% of participants complete the study while attending ≥75% of acupuncture sessions.

### Secondary outcomes

#### Feasibility

Consent rate, patient and parent satisfaction (measured at weeks 6, 10 and 14 via a 5-point Likert scale), and assessment of required personnel capacity for a future RCT (measured at end of study via open feedback by involved staff members on feasibility and workload).

#### Acupuncture points

Documentation of targeted acupuncture points.

#### Safety

Occurrence of AEs (needle-related pain, irritation, haematomas, headache, syncope, sedation, neuropathy) assessed at weeks 3, 6, 10 and 14. Severity and treatment relation will be recorded.

#### Efficacy

Treatment success, defined as resolution of FC per Rome IV criteria at the end of the intervention (EOI).^3 37^ Rome IV criteria will be assessed by the investigator at the screening visit (visit 1) and at the EOI visit. For the EOI assessment, the criteria will be assessed over the last 4 weeks of the acupuncture intervention, or, in the event a participant discontinues the study prematurely within 4 weeks, over the intervention period. Other efficacy endpoints will be assessed via symptom diaries, the endpoints, including quality of life and school absence, are based on a previously published core outcome set^45^:

- Defecation frequency (bowel movements/week).
- Stool consistency (Bristol Stool Scale).
- Painful defecation (yes/no).
- Faecal incontinence frequency (episodes/week).
- Withholding behaviour (yes/no).
- Abdominal pain (yes/no, Visual Analogue Scale score).
- Spontaneous bowel movements (>24 hours after escape medication).
- Time between acupuncture and defecation.
- Bowel movements <24 hours after acupuncture.
- Escape medication use and effect.

#### Quality of life

Assessed at weeks 0, 6, 10 and 14 using PedsQL 4.0 Generic Core Scale and PedsQL 3.0 Gastrointestinal Symptoms Scale.^46 47^

#### School absence

Monitored via a self-designed questionnaire.

#### Anthropometrics

Body weight (kg) and height (cm) measured at baseline and week 14.

### Statistical analysis

Continuous data will be presented as mean±SD if normally distributed and as median with IQR otherwise. Categorical data will be presented as proportions. The 95% CI will be calculated for all primary and secondary parameters, with significance set at α=0.05. The primary outcome, attrition rate, is defined as the proportion of participants completing the study. Attendance at 100%, ≥75% and ≥50% of acupuncture sessions will be reported. Feasibility is confirmed if ≥70% of participants complete the study while attending ≥75% of sessions, informing a future RCT design. Secondary feasibility outcomes include consent rate, satisfaction and personnel capacity. Consent rate is the proportion of approached participants who provided consent, with age distribution reported as mean±SD or median (IQR). Reasons for non-participation will be described. Satisfaction scores (5-point Likert scale) at weeks 6, 10 and 14 will be summarised, visualised with bar plots and analysed descriptively. Personnel requirements will be summarised.

A table will summarise acupuncture points. Safety analysis includes AE frequency. Efficacy, assessed as the proportion no longer meeting Rome IV criteria, will be analysed using McNemar’s test. Due to the study design, no efficacy claims can be made without a control group.

### Interim analysis

An interim analysis will be conducted after 10 participants have completed the study, including follow-up, to evaluate feasibility. This unblinded analysis will assess attrition rate, consent rate, patient and parent satisfaction, and retention rate. The interim findings will be discussed with the research team to determine whether the study should continue as planned. The principal investigator will make the final decision regarding trial continuation and will report this decision to the METC.

### Ethics and dissemination

#### Ethics approval

Ethics approval was granted by the METC of the Amsterdam UMC (NL87083.018.24). This study will be conducted according to the principles of the Declaration of Helsinki (64th WMA General Assembly, Fortaleza, Brazil, October 2013) and in accordance with the Medical Research Involving Human Subjects Act.

#### Registration details

ClinicalTrials.gov (NCT06836362) and CCMO (NL-OMON57236).

#### Dissemination

The results of this pilot study will be presented at national and international conferences and published in an international medical journal.

## Supplementary material

10.1136/bmjopen-2025-109425 online supplemental file 1

## References

[1] Koppen IJN, Vriesman MH, Saps M (2018). Prevalence of Functional Defecation Disorders in Children: A Systematic Review and Meta-Analysis. J Pediatr.

[2] Koppen IJN, Lammers LA, Benninga MA (2015). Management of Functional Constipation in Children: Therapy in Practice. Paediatr Drugs.

[3] Hyams JS, Di Lorenzo C, Saps M (2016). Functional Disorders: Children and Adolescents. Gastroenterology.

[4] Varni JW, Bendo CB, Nurko S (2015). Health-Related Quality of Life in Pediatric Patients with Functional and Organic Gastrointestinal Diseases. J Pediatr.

[5] Vriesman MH, Rajindrajith S, Koppen IJN (2019). Quality of Life in Children with Functional Constipation: A Systematic Review and Meta-Analysis. J Pediatr.

[6] Copeland WE, Wolke D, Angold A (2013). Adult Psychiatric Outcomes of Bullying and Being Bullied by Peers in Childhood and Adolescence. JAMA Psychiatry.

[7] Baker SS, Liptak GS, Colletti RB (1999). Constipation in Infants and Children: Evaluation and Treatment. J Pediatr Gastroenterol Nutr.

[8] Liem O, Harman J, Benninga M (2009). Health Utilization and Cost Impact of Childhood Constipation in the United States. J Pediatr.

[9] Park R, Mikami S, LeClair J (2015). Inpatient burden of childhood functional GI disorders in the USA: an analysis of national trends in the USA from 1997 to 2009. Neurogastroenterology Motil.

[10] Tabbers MM, DiLorenzo C, Berger MY (2014). Evaluation and treatment of functional constipation in infants and children: evidence-based recommendations from ESPGHAN and NASPGHAN. J Pediatr Gastroenterol Nutr.

[11] Koppen IJN, van Wassenaer EA, Barendsen RW (2018). Adherence to Polyethylene Glycol Treatment in Children with Functional Constipation Is Associated with Parental Illness Perceptions, Satisfaction with Treatment, and Perceived Treatment Convenience. J Pediatr.

[12] Vriesman MH, Koppen IJN, Camilleri M (2020). Management of functional constipation in children and adults. *Nat Rev Gastroenterol Hepatol*.

[13] Vlieger AM, Blink M, Tromp E (2008). Use of complementary and alternative medicine by pediatric patients with functional and organic gastrointestinal diseases: results from a multicenter survey. Pediatrics.

[14] Li H, He T, Xu Q (2015). Acupuncture and regulation of gastrointestinal function. *WJG*.

[15] Birch S (2016). Shonishin: Japanese Pediatric Acupuncture.

[16] Wang L, Xu M, Zheng Q (2020). The Effectiveness of Acupuncture in Management of Functional Constipation: A Systematic Review and Meta‐Analysis. Evid Based Complement Alternat Med.

[17] Wang X, Wang H, Guan Y (2021). Acupuncture for functional gastrointestinal disorders: A systematic review and meta‐analysis. J of Gastro and Hepatol.

[18] Yu Z (2020). Neuromechanism of acupuncture regulating gastrointestinal motility. WJG.

[19] Carabotti M, Scirocco A, Maselli MA (2015). The gut-brain axis: interactions between enteric microbiota, central and enteric nervous systems. Ann Gastroenterol.

[20] Bonaz B, Sinniger V, Pellissier S (2017). The Vagus Nerve in the Neuro-Immune Axis: Implications in the Pathology of the Gastrointestinal Tract. Front Immunol.

[21] Bonaz B, Sinniger V, Pellissier S (2021). Therapeutic Potential of Vagus Nerve Stimulation for Inflammatory Bowel Diseases. Front Neurosci.

[22] Chang Y-M, El-Zaatari M, Kao JY (2014). Does stress induce bowel dysfunction?. Expert Review of Gastroenterology & Hepatology.

[23] Guo J, Chen L, Wang Y (2022). Electroacupuncture Attenuates Post-Inflammatory IBS-Associated Visceral and Somatic Hypersensitivity and Correlates With the Regulatory Mechanism of Epac1–Piezo2 Axis. Front Endocrinol.

[24] Chu D, Cheng P, Xiong H (2011). Electroacupuncture at ST-36 relieves visceral hypersensitivity and decreases 5-HT3 receptor level in the colon in chronic visceral hypersensitivity rats. Int J Colorectal Dis.

[25] Kotani N, Hashimoto H, Sato Y (2001). Preoperative Intradermal Acupuncture Reduces Postoperative Pain, Nausea and Vomiting, Analgesic Requirement, and Sympathoadrenal Responses. Anesthesiology.

[26] Lee I-S, Cheon S, Park J-Y (2019). Central and Peripheral Mechanism of Acupuncture Analgesia on Visceral Pain: A Systematic Review. Evid Based Complement Alternat Med.

[27] Wang M, Liu W, Ge J (2023). The immunomodulatory mechanisms for acupuncture practice. Front Immunol.

[28] Du L, Qin Q, He X (2025). Interstitial Cells of Cajal Are Required for Different Intestinal Motility Responses Induced by Acupuncture. *Neurogastroenterol Motil*.

[29] Guo J, Yang X, Yang J (2025). Electroacupuncture Promotes the Proliferation and Differentiation of Enteric Neural Precursor Cells via the PTEN/PI3K/Akt/mTOR Signaling Pathway in Diabetic Mice. *Neurogastroenterol Motil*.

[30] Langevin HM, Bouffard NA, Badger GJ (2006). Subcutaneous tissue fibroblast cytoskeletal remodeling induced by acupuncture: Evidence for a mechanotransduction‐based mechanism. *Journal Cellular Physiology*.

[31] Langevin HM, Churchill DL, Fox JR (2001). Biomechanical response to acupuncture needling in humans. J Appl Physiol.

[32] Broide E, Pintov S, Portnoy S (2001). Effectiveness of Acupuncture for Treatment of Childhood Constipation. Dig Dis Sci.

[33] Anders EF, Findeisen A, Nowak A (2012). Acupuncture for treatment of hospital-induced constipation in children: a retrospective case series study. Acupunct Med.

[34] Abd El Azeem AM, Alsharnoubi J, Abd El-Rahman Mohamed M (2023). Laser acupuncture improving functional chronic constipation in children: a randomized controlled trial. Lasers Med Sci.

[35] Serra J, Pohl D, Azpiroz F (2020). European society of neurogastroenterology and motility guidelines on functional constipation in adults. *Neurogastroenterol Motil*.

[36] Liu B, Wu J, Yan S (2021). Electroacupuncture vs Prucalopride for Severe Chronic Constipation: A Multicenter, Randomized, Controlled, Noninferiority Trial. *Am J Gastroenterol*.

[37] Koppen IJN, Saps M, Lavigne JV (2018). Recommendations for pharmacological clinical trials in children with functional constipation: The Rome foundation pediatric subcommittee on clinical trials. *Neurogastroenterology Motil*.

[38] MacPherson H, Altman DG, Hammerschlag R (2010). Revised Standards for Reporting Interventions in Clinical Trials of Acupuncture (Stricta): Extending the Consort Statement. Acupunct Med.

[39] Viechtbauer W, Smits L, Kotz D (2015). A simple formula for the calculation of sample size in pilot studies. J Clin Epidemiol.

[40] Whitehead AL, Julious SA, Cooper CL (2016). Estimating the sample size for a pilot randomised trial to minimise the overall trial sample size for the external pilot and main trial for a continuous outcome variable. Stat Methods Med Res.

[41] Mugie SM, Korczowski B, Bodi P (2014). Prucalopride is no more effective than placebo for children with functional constipation. Gastroenterology.

[42] Benninga MA, Hussain SZ, Sood MR (2022). Lubiprostone for Pediatric Functional Constipation: Randomized, Controlled, Double-Blind Study With Long-term Extension. Clin Gastroenterol Hepatol.

[43] Adams D, Cheng F, Jou H (2011). The safety of pediatric acupuncture: a systematic review. *Pediatrics*.

[44] Yang C, Hao Z, Zhang LL (2015). Efficacy and safety of acupuncture in children: an overview of systematic reviews. *Pediatr Res*.

[45] Kuizenga-Wessel S, Steutel NF, Benninga MA (2017). Development of a core outcome set for clinical trials in childhood constipation: a study using a Delphi technique. BMJ Paediatr Open.

[46] Varni JW, Bendo CB, Denham J (2014). PedsQL gastrointestinal symptoms module: feasibility, reliability, and validity. *J Pediatr Gastroenterol Nutr*.

[47] Varni JW, Burwinkle TM, Seid M (2003). The PedsQL 4.0 as a pediatric population health measure: feasibility, reliability, and validity. Ambul Pediatr.

