# Supplementary material for: Prospective, non-randomised, open-label pilot trial assessing feasibility, safety and treatment success of acupuncture in children with functional constipation: ACU-PILOT study protocol
Source: BMJ Open. 2025 Nov 11;15(11):e109425. doi: 10.1136/bmjopen-2025-109425 (PMC12606485; doi:10.1136/bmjopen-2025-109425)
Supplement: online supplemental file 1 [file bmjopen-15-11-s001.docx]

**Appendices AcuPilot**

**Figure supplement 1:**

**Acupuncture treatment decision tree according to age groups – sessions 2 to 4**

**
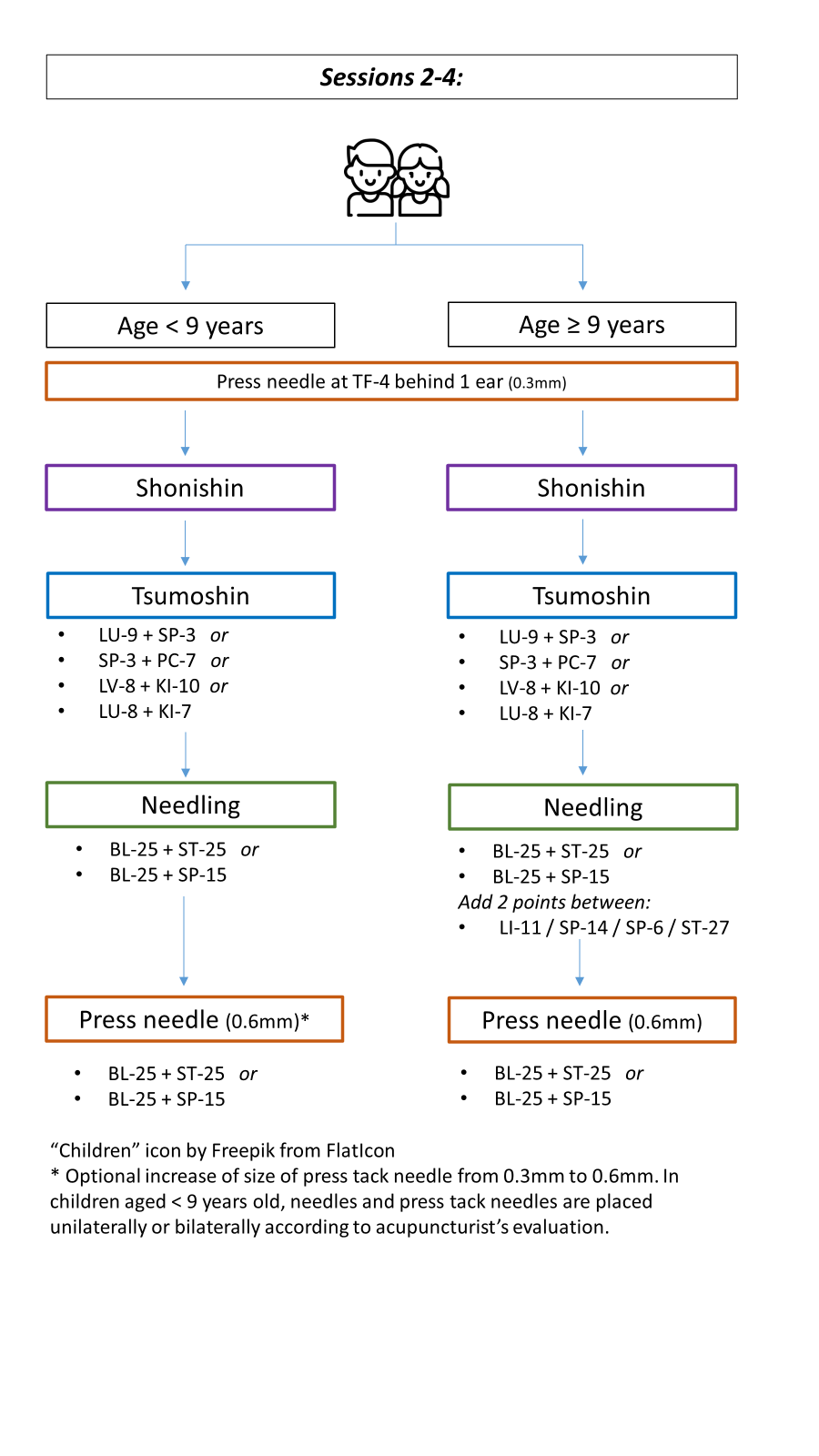
**

**Figure supplement 2:**

**Acupuncture treatment decision tree according to age groups – sessions 5-8**

**
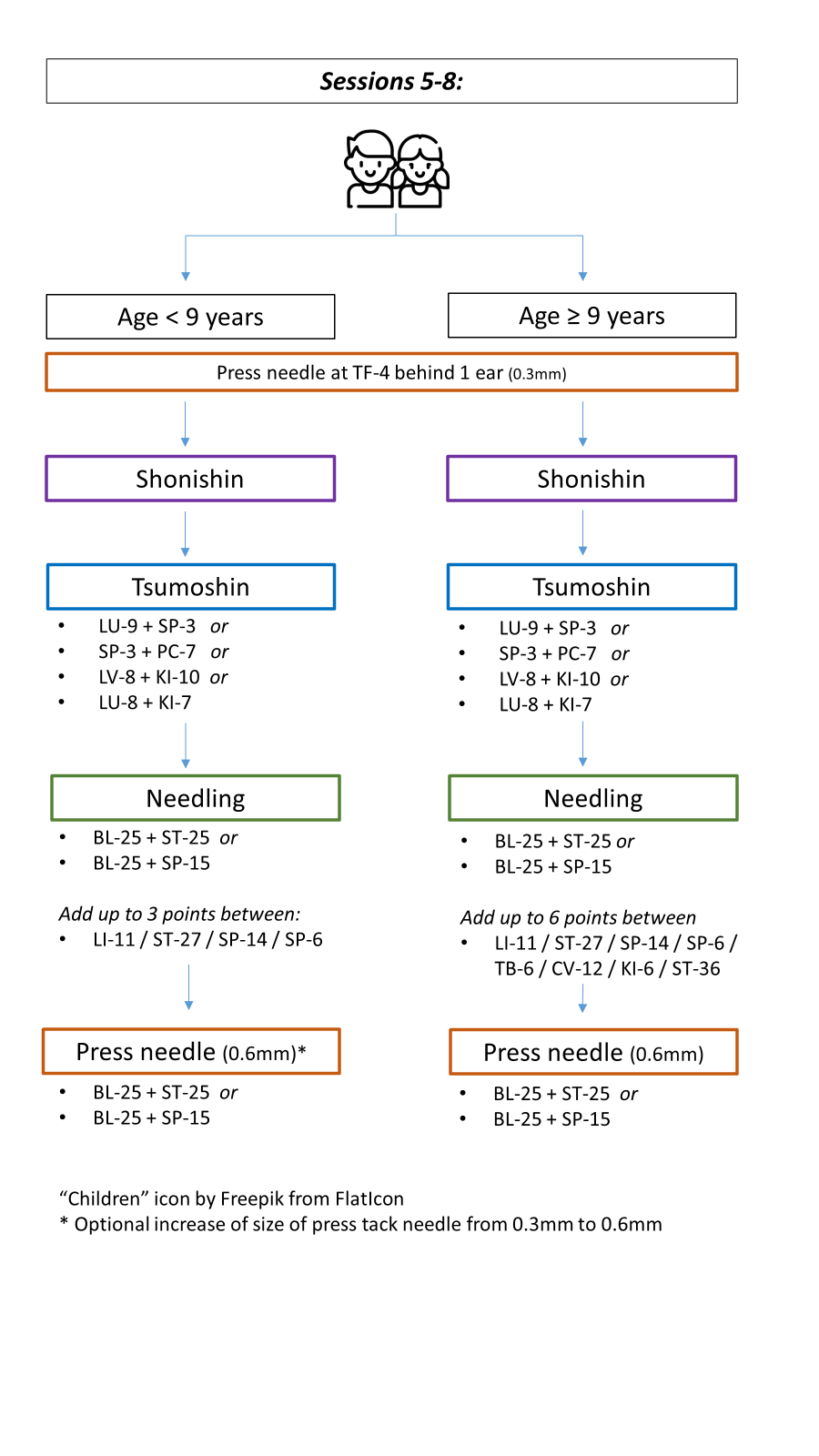
**

**Appendix 1: Basic Shonishin treatment pattern**

**
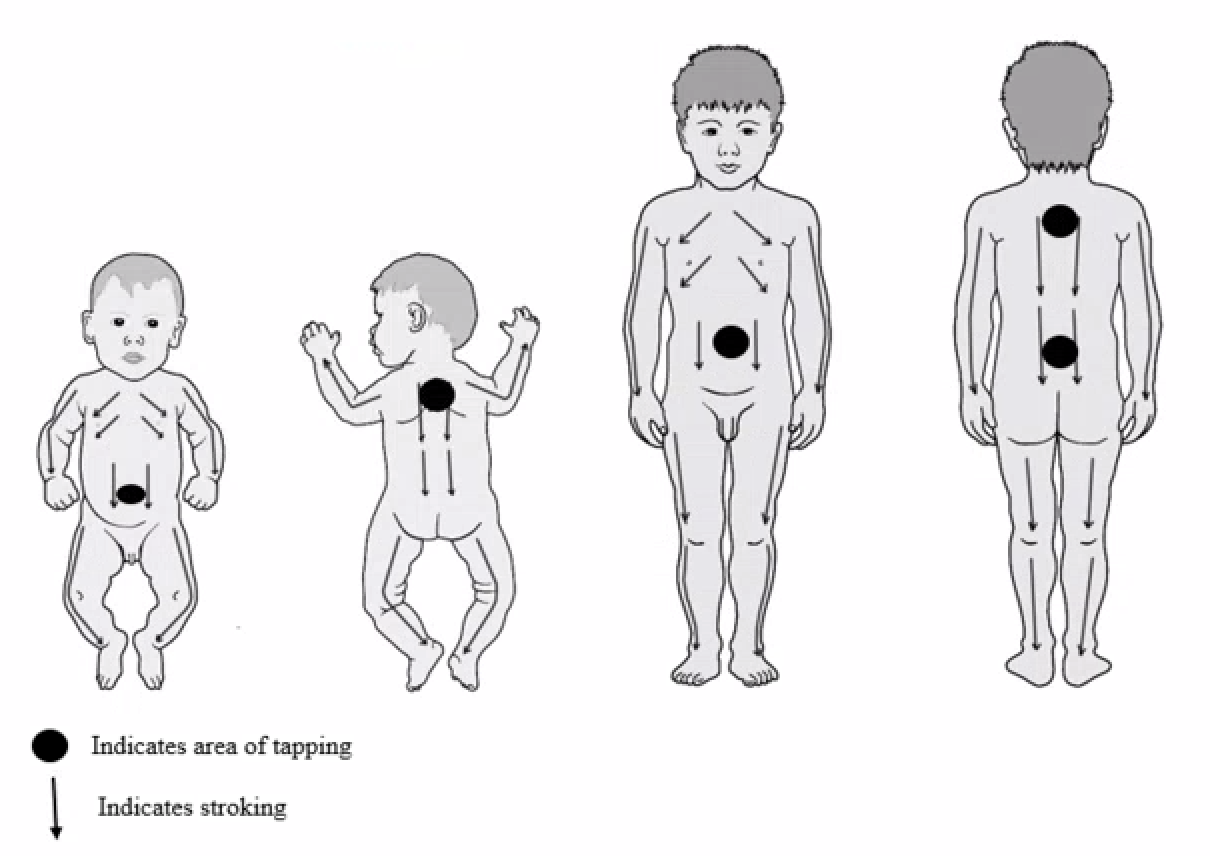
**

*Shonishin – a Japanese rendering of the older Chinese term* erzhen, *which translates to “children’s needle” or “children’s needling”.^1^ Stroking is applied with light pressure down the back and back of the legs, down the abdomen and anterio-lateral edges of the legs and down the arms. Tapping is applied around T2-T4, around L3-5 and around the umbilicus. The number of strokes and taps and force of contact are positively correlated to increased child’s age. If the child has problems with eczema or atopic dermatitis, stroking is not used, instead tapping is applied to the acupoints: LI-4, LI-10, LI-11, LI-15, GV-12, GV-3, SP-10, BL-40, ST-36. ^1^*

**Appendix 2: Signs and symptoms of the 4 patterns of Meridian Therapy diagnosis in children**

The Tsumoshin tool is a blunt-tipped non-penetrating needle with a rounded millet-seed-like point used for pressing the body surface. As part of the standard intervention, children will be treated with the tsumoshin tool with light pressure on a combination of two points, based on the root pattern (see below pattern / acupoints). The choice of the root pattern treatment is determined by the acupuncturist. This choice is based on factors considered to contribute to defecatory problems according to Traditional Japanese Medicine after medical history and physical examination^1^

- Lung pattern
  - Acupoints LU-9 + SP-3 (If SP-3 is too ticklish for the child SP-5 is used instead)
  - Constipation with history of dermatologic and/or respiratory problems (atopic diseases)
- Spleen pattern
  - Acupoints SP-3 + PC-7
  - Constipation with predominance of gastrointestinal symptoms (abdominal pain, bloating, reduced appetite) and absence of other pattern predominance
- Kidney pattern
  - Acupoints KI-7 + LU-8 (or KI-10 + LU-5 in case of bedwetting in children > 7 years old)
  - Constipation with predominance of urological symptoms (bedwetting) and/or problems in motor development skills
- Liver pattern
  - Acupoints LV-8 + KI-10
  - Constipations with predominance of behavioural problems (irritability, hyperactivity)

**Appendix 3: Acupoint locations**

Meridian Therapy treatment points:

- **LU-9** is at the juncture of the medial edge of the abductor pollicis longus tendon on the wrist crease, between the tendon and radial artery
- **LU-8** is along medial edge of the tendon of the abductor pollicis longus level with the high point of the styloid process
- **LU-5** is on the radial edge of the brachial artery on the elbow crease.
- **PC-7** is between the tendons of musculus palmaris longus and flexor carpi radialis on the wrist crease
- **KI-7** is about 2 cun* above the level of KI-3 along the anterior margin of the Achilles tendon.
- **SP-3** is in a depression on the lower margin of the abductor hallucis tendon at the proximal edge of the distal head of the 1^st^ meta-tarsal bone.
- **SP-5** is in the depression in front of the medial malleolus level with the anterior and inferior margins of the malleolus
- **KI-10** is at the juncture of the popliteal fossa and posterior margin of the Sartorius muscle.
- **LV-8** is on the anterior margin of the Sartorius muscle. It is on the line from the midpoint of the patella and the finger at KI-10 as that line intersects the anterior margin of the Sartorius muscle.

Stress-reduction point:

- **TF-4** (Behind ShenMen) is located on the back of the ear directly behind the point ShenMen on the anterior aspect at the bifurcation of the crura of the antihelix. This point is found by visual examination.

Constipation treatment points:

- **ST-25** is 2 cun* lateral to the midpoint of the navel, either side of the navel in the rectus abdominis muscle
- **BL-25** is 1.5 cun* from the midline lateral to the lower margin of lumbar 4, either side of the spine
- **SP-15** is 4 cun* lateral to the center of the navel
- **ST-36** is located on the line one third the distance from the most distal edge of the tibial tuberosity going towards the lower margin of the lateral head of the fibula.
- **LI-11** bending the arm, the point is at the lateral end of the elbow crease
- **ST-27** is 2 cun* lateral to the midline of the abdomen, 2/5 of the distance from the navel to the pubis symphysis
- **SP-6** is on the medial aspect of the lower leg, 3 cun* above the medial malleolus, on the posterior border of the medial aspect of the tibia.
- **KI-6** is on the medial aspect of the foot, in the depression below the tip of the medial malleolus.
- **SJ-6** is on the dorsal aspect of the forearm, on the line connecting SJ 4 and the tip of the elbow, 3 cun* above the transverse crease of the wrist between the ulna and radius.
- **SP-14** is 1.3 cun* below SP-15 and 4 cun lateral from the midline
- **CV-12** is on the midline, 4 cun* superior to the umbilicus.

**1 cun is a traditional Chinese body measure that is approximately 2.3 cm in length*


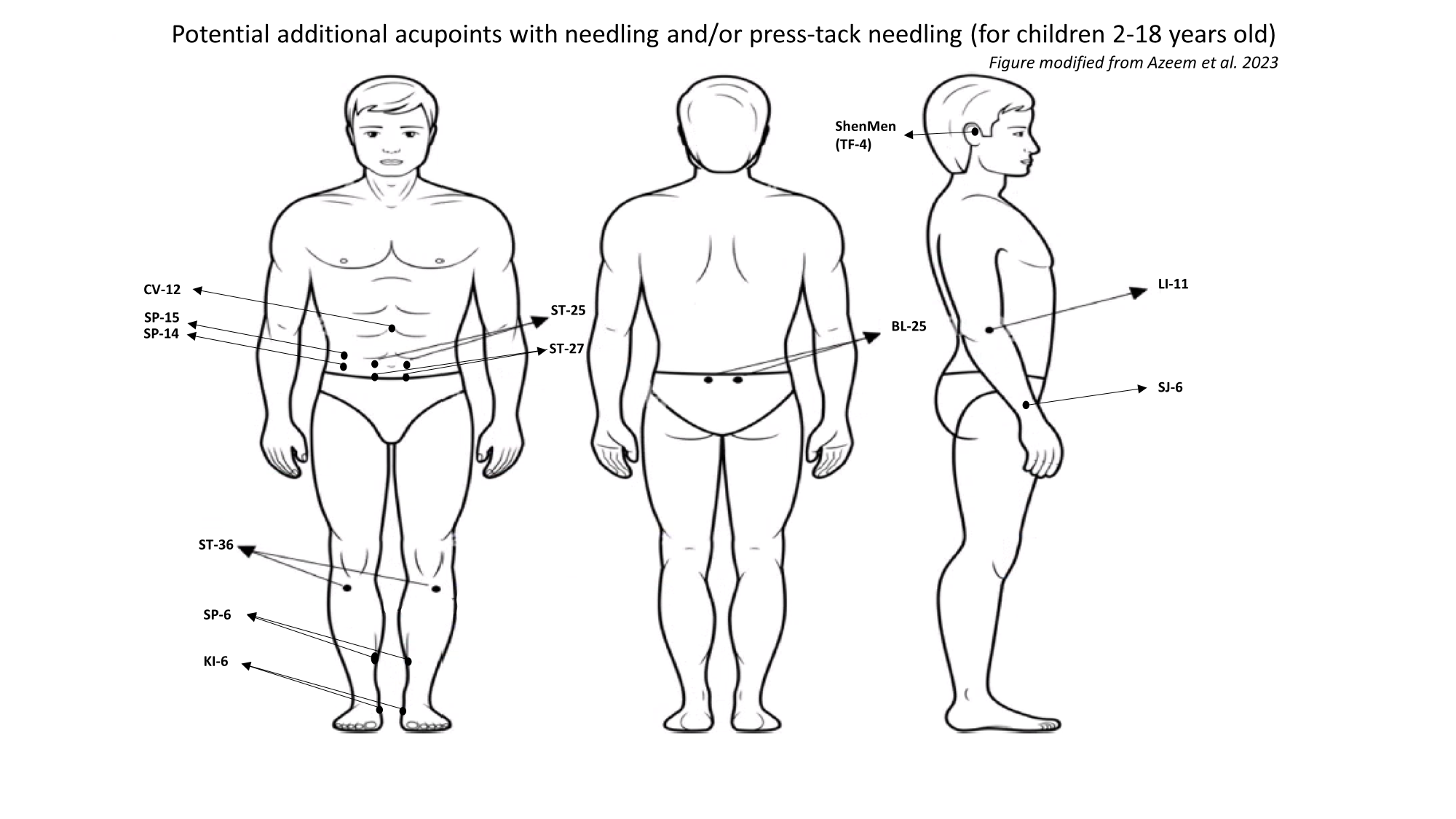


^2^

**References**

1. Birch S. *Shonishin: Japanese Pediatric Acupuncture*. vol Second Edition. Thieme; 2016:346.

2. El Azeem AMA, Alsharnoubi JA-O, Abd El-Rahman Mohamed M. Laser acupuncture improving functional chronic constipation in children: a randomized controlled trial. 2023;(1435-604X (Electronic))
