# Supplementary material for: Prospective, non-randomised, open-label pilot trial assessing feasibility, safety and treatment success of acupuncture in children with functional constipation: ACU-PILOT study protocol
Source: BMJ Open. 2025 Nov 11;15(11):e109425. doi: 10.1136/bmjopen-2025-109425 (PMC12606485; doi:10.1136/bmjopen-2025-109425)
Supplement: Supplementary file 2 [file bmjopen-2025-109425.reviewer_comments.pdf]

PEER REVIEW HISTORY

BMJ Open publishes all reviews undertaken for accepted manuscripts. Reviewers are asked to complete a checklist review form and are provided with free text boxes to elaborate on their assessment. These free text comments are reproduced below.

ARTICLE DETAILS

Title (Provisional)

A prospective, non-randomized, open-label pilot trial assessing feasibility, safety and treatment success of acupuncture in children with functional constipation: ACU-PILOT study protocol

Authors

Bloem, Michelle N.; Baaleman, Desiree; Koppen, Ilan J.N.; De Lorijn, Fleur; Vlieger, Arine M.; Birch, Stephen; nieuwdorp, max; Benninga, Marc A.

VERSION 1 - REVIEW

**Reviewer**	**1**
**Name**	**Xiao, Xianjun**
**Affiliation**	**Chengdu University of Traditional Chinese Medicine**
**Date**	**24-Sep-2025**
**COI**	**None**

This is a well-designed pilot study. Its core objective is to assess the feasibility and safety of acupuncture for pediatric FC and provide design rationale for future large-scale RCT. This positioning is accurate and pragmatic. However, several limitations warrant attention:

1. Although the authors mention this is a pilot study, the complete absence of a control group may lead to confounding from placebo effects and natural disease course improvement.

2. The abstract and main text both mention this is a multicenter study, but the participating institutions are not presented in the Recruitment procedure section.

3. In Visit 2 of the Study schedule, what is the purpose of bowel cleanout? Is this necessary for children? Particularly considering that the study population consists of children, bowel cleanout may increase patient burden and compliance issues.

4. Regarding adverse event assessment, the current plan includes evaluations at weeks 3, 6, and 10. It is recommended to add a follow-up timepoint at week 8, as the extended intervals may introduce recall bias.

5. In Treatment progresses, why are different acupuncture intervention protocols used across different sessions, and what is the purpose?

6. I do not fully understand the "Consent rate" - does this refer to the proportion of initially screened patients who agree to enrollment?

**Reviewer**	**2**
**Name**	**Wu, Huangan**
**Affiliation**	**Shanghai University of Traditional Chinese Medicine**
**Date**	**25-Sep-2025**
**COI**	**None**

The authorship reflects a strong multidisciplinary collaboration between pediatric gastroenterologists, acupuncture experts, and clinical trial methodologies.

Minor Revisions:
1. While the protocol is detailed, the flexibility in acupoint selection based on pattern diagnosis, while clinically realistic, may reduce reproducibility. I recommend including a minimum core set of points required at each session to improve consistency across practitioners and future trials.
2. Given the exploratory nature, incorporating objective biomarkers (e.g., heart rate variability, stool microbiota, or serum biomarkers related to gut-brain axis modulation) could provide preliminary insights into acupuncture's mechanism in children—a valuable addition for a future RCT.

VERSION 1 - AUTHOR RESPONSE

Reviewer: 1

Dr. Xianjun Xiao, Chengdu University of Traditional Chinese Medicine, Wenjiang District Chinese Medicine Hospital

Comments to the Author:

This is a well-designed pilot study. Its core objective is to assess the feasibility and safety of acupuncture for pediatric FC and provide design rationale for future large-scale RCT. This positioning is accurate and pragmatic.

➔ Thank you for your thoughtful comment and for recognizing the design and objectives of our study.

However, several limitations warrant attention:

1. Although the authors mention this is a pilot study, the complete absence of a control group may lead to confounding from placebo effects and natural disease course improvement.

➔ Thank you for your comment. As this is primarily a feasibility study, our main aim is to assess the practicality, safety, and acceptability of acupuncture for pediatric functional constipation, and to gather essential data to inform the design of a future large-scale RCT in children with functional constipation. The absence of a control group at this stage is intentional, as our focus is on feasibility outcomes rather than efficacy. Once feasibility has

been established, we plan to include a control group in the subsequent RCT to address potential confounding factors and the natural course of the disease. This approach was discussed extensively with our Medical Ethics Committee, who approved the study design as appropriate for this initial phase.

2. The abstract and main text both mention this is a multicenter study, but the participating institutions are not presented in the Recruitment procedure section.

- ➔ Thank you for highlighting this. While the protocol was designed to allow for a multicenter study, it is now expected that recruitment will proceed rapidly and the study will most likely be conducted as a monocenter study. We have corrected the abstract and main text to reflect this.

3. In Visit 2 of the Study schedule, what is the purpose of bowel cleanout? Is this necessary for children? Particularly considering that the study population consists of children, bowel cleanout may increase patient burden and compliance issues.

- ➔ Thank you for your question regarding the purpose of bowel cleanout at Visit 2. The bowel cleanout (fecal desimpaction) is included to standardize the baseline situation across all participating children, ensuring that each child starts the intervention phase with a similar bowel status. This approach is recommended in international guidelines for the management of pediatric functional constipation, including the recommendations from the Rome Foundation Pediatric Subcommittee (Koppen et al., 2018). These guidelines emphasize the importance of standardizing baseline conditions in clinical trials, as well as the use of fecal desimpaction prior to intervention, to optimize the effectiveness of subsequent treatments and allow for more reliable assessment of outcomes. We recognize that bowel cleanout may increase patient burden and could potentially affect compliance, especially in a pediatric population. However, by following these guideline recommendations and standardizing the initial condition, we aim to enhance the validity and comparability of our study results. In addition, in cases of a full rectum, it is often not possible to achieve adequate bowel evacuation with oral medication alone. For this reason, we considered it important to include bowel cleanout in the context of this acupuncture study. We will closely monitor and document any issues related to patient burden and compliance as part of our feasibility assessment.

  Reference: Koppen IJN, Saps M, Lavigne JV, Nurko S, Taminiau JAJM, Di Lorenzo C, Benninga MA. Recommendations for pharmacological clinical trials in children with functional constipation: The Rome foundation pediatric subcommittee on clinical trials. Neurogastroenterol Motil. 2018 Apr;30(4):e13294. doi: 10.1111/nmo.13294. Epub 2018 Jan 30. PMID: 29380480.

4. Regarding adverse event assessment, the current plan includes evaluations at weeks 3, 6, and 10.

It is recommended to add a follow-up timepoint at week 8, as the extended intervals may introduce recall bias.

➔ Thank you for your suggestion regarding adverse event assessment. At each study visit, patients are asked about side effects, and they are encouraged to report any adverse events via email at any time throughout the study period. In addition to this ongoing monitoring, we have scheduled four fixed timepoints (weeks 3, 6, and 10 and 14) for more extensive evaluation of side effects. We believe this approach allows for both continuous and structured assessment of adverse events, while balancing participant burden. However, we appreciate your recommendation.

5. In Treatment progresses, why are different acupuncture intervention protocols used across different sessions, and what is the purpose?

➔ The acupuncture intervention protocol is structured into multiple sessions with a stepwise approach, and a clear subdivision is made between children under 9 years and those 9 years and older. In the first session, younger children (<9 years) receive only non-penetrating Shonishin techniques and press-tack needles, while older children (≥9 years) receive a combination of press-tack and inserted needles. For all participants, step-ups in the number and type of acupuncture points are introduced at sessions 2 and 5, forming the basis of our semi-standardized protocol.

This semi-standardized approach was developed to balance clinical reproducibility with the individualized principles of pediatric acupuncture. Starting with fewer acupuncture points in the initial session allows children (especially young children) to become accustomed to the procedure. As treatment progresses, additional points are incorporated based on the child's age, clinical response, and evolving pattern diagnosis, reflecting clinical acupuncture practice. The protocol combines a core set of required acupoints with optional points selected from a predefined set, based on literature review, expert consensus, and a survey among experienced pediatric acupuncturists. This design allows for both standardization and individualized care, and all modifications or step-ups are carefully documented.

The purpose of this approach is to make the treatment approachable for children, to maintain a clear and reliable study design, and to find out whether this individualized acupuncture method is practical and acceptable for use in children in a research setting.

6. I do not fully understand the "Consent rate" - does this refer to the proportion of initially screened patients who agree to enrollment?

➔ Thank you for your question. We have defined this on page 13 as follows: "Consent rate is the proportion of approached participants who provided consent, with age distribution reported as mean ± SD or median (IQR). Reasons for non-participation will be described." The consent rate refers to the percentage of eligible patients (and/or their parents/guardians) who, after being approached for participation, agree to be enrolled in the study.

Reviewer: 2
Prof. Huangan Wu, Shanghai University of Traditional Chinese Medicine

Minor Revisions:

1. While the protocol is detailed, the flexibility in acupoint selection based on pattern diagnosis, while clinically realistic, may reduce reproducibility. I recommend including a minimum core set of points required at each session to improve consistency across practitioners and future trials.

- ➔ Thank you for your suggestion. We agree with your point and have included a minimum core set of acupoints at each session, which is expanded in subsequent sessions. Specifically, this core set depends on the patient's age category (<9 years or 9 years and older). For all children, the minimum core set consists of non-penetrating Shonishin and Tsumoshin techniques, a press-tack needle at TF-4 (ShenMen), and press-tack needles at BL-25 and either ST-25 or SP-15. In older children, bilateral needling at BL-25 and ST-25 or SP-15 is also included. As treatment progresses, additional points are incorporated in a stepwise manner based on clinical response and pattern diagnosis, resulting in a semi-standardized protocol (pp.10-11). We believe this approach enhances reproducibility while still allowing for the principles of individualized acupuncture treatment.

  We also have updated our Strength and Limitations bullet points (p.2) to reflect this point, by adding the sentence: "Acupuncture is administered according to a semi-standardized protocol, combining a core set of acupoints with individualized point selection based on clinical assessment."

2. Given the exploratory nature, incorporating objective biomarkers (e.g., heart rate variability, stool microbiota, or serum biomarkers related to gut-brain axis modulation) could provide preliminary insights into acupuncture's mechanism in children—a valuable addition for a future RCT.

- ➔ We agree with your recommendation and recognize the value of incorporating objective biomarkers in future studies. In fact, we initially included the collection of fecal samples and other biomarkers in our protocol. However, after discussion with our Medical Ethics Committee (METC), it was advised that such procedures would be too invasive for a feasibility study in this pediatric population. We therefore decided to postpone the collection of these samples until the subsequent randomized controlled trial, where we plan to include fecal samples for microbiota analysis and consider additional biomarkers such as heart rate variability and serum markers related to gut-brain axis modulation.

Comments to the Author:
The authorship reflects a strong multidisciplinary collaboration between pediatric gastroenterologists, acupuncture experts, and clinical trial methodologies.

- ➔ Thank you
